# Free-Floating Thrombus in Stroke: Therapeutic Dilemmas and Case Series Analysis

**DOI:** 10.7759/cureus.89156

**Published:** 2025-07-31

**Authors:** Hajar Khattab, Manal Khalfi, Kamal Haddouali, Asmae Sikkal, Salma Bellakhdar, Hicham El Otmani, Bouchra El Moutawakil, Mohammed Abdoh Rafai

**Affiliations:** 1 Department of Neurology, Ibn Rochd University Hospital, Casablanca, MAR; 2 Department of Neurology, Faculty of Medicine and Pharmacy of Casablanca, Hassan II University, Casablanca, MAR; 3 Research Laboratory on Diseases of the Nervous System, Neurosensory and Handicap, Faculty of Medicine and Pharmacy of Casablanca, Hassan II University, Casablanca, MAR; 4 Department of Neurology and Neurophysiological Explorations, Ibn Rochd University Hospital, Casablanca, MAR; 5 Laboratory of Genetics and Molecular Pathology, Faculty of Medicine and Pharmacy of Casablanca, Hassan II University, Casablanca, MAR

**Keywords:** antithrombotics, atheroma, carotid web, endarterectomy, free-floating thrombus, stroke

## Abstract

Background and aims: Free-floating thrombus (FFT) is a rare but clinically significant vascular finding, not only in ischemic stroke but also in other contexts such as routine imaging for asymptomatic patients or evaluation of other vascular diseases. It is characterized by an intraluminal thrombus adherent to the arterial wall with partial luminal occlusion and cyclic movement synchronized with the cardiac cycle. Although associated with an increased risk of embolic complications, including recurrence, no consensus exists regarding optimal management. This study aims to describe the clinical and radiological characteristics of FFTs in a hospital-based case series and to report treatment strategies and outcomes.

Methods: A retrospective study was conducted over a 17-month period (2023-2024) at Ibn Rochd University Hospital, including patients with acute ischemic stroke and FFT diagnosed via computed tomography angiography (CTA) and/or duplex ultrasound (DUS). Collected data included age, NIHSS score at admission, etiological workup, and both clinical and imaging outcomes. Therapeutic management was guided by stroke severity, hemorrhagic risk, availability of treatment modalities, and the results of the etiological workup.

Results: Among 940 ischemic stroke cases, FFT was identified in eight patients (0.8%), with a mean age of 50.6 years. Atherosclerosis was the predominant etiology. The thrombus was most frequently localized in the carotid arteries, but also involved the brachiocephalic and vertebral arteries. No patients received intravenous thrombolysis due to concerns regarding embolization risk. Medical management was applied in all cases, with six patients receiving anticoagulation therapy, and five of them receiving combined antiplatelet therapy and statins. One patient underwent delayed carotid endarterectomy following stroke recurrence. At three months, thrombus resolution was complete in four cases, while the remaining patients exhibited partial regression.

Conclusion: FFT represents a high-risk entity for stroke recurrence, requiring individualized management. In our limited case series, medical management (primarily anticoagulation, sometimes combined with antiplatelet therapy) was observed to be associated with thrombus resolution in most patients. Delayed surgical or endovascular intervention may be considered in cases of persistent thrombus or recurrence. These observations should be interpreted with caution. Larger prospective studies are needed to define optimal management strategies and clarify the role of endovascular interventions.

## Introduction

Free-floating thrombus (FFT), also known as intraluminal thrombus, is a rare but increasingly recognized emergent marker in ischemic stroke [1]. It is characterized by a thrombus adherent to the arterial wall with circumferential blood flow around its distal portion, often displaying cyclic movement synchronized with the cardiac cycle [2]. The reported prevalence of FFT in acute ischemic stroke ranges from 1.6% to 3.2%, with a higher incidence (14.3%) in patients with high-grade carotid stenosis [3]. Despite being first documented in the 1960s, FFT remains a poorly understood entity due to its similarities with embolic thrombi and mobile plaques, which complicate its definition and classification.

The etiology of FFT is diverse, with atherosclerosis being the most common underlying cause [4]. However, other conditions such as arterial dissection, carotid web, vasculitis, malignancies, hypercoagulable states, and infectious or inflammatory diseases have also been implicated. The shift from digital subtraction angiography (DSA) to non-invasive imaging techniques, particularly computed tomography angiography (CTA) and carotid duplex ultrasound (DUS), has improved the detection and follow-up of FFT [5]. 

FFT can occur in various locations within the cervicocranial arterial system, with a higher prevalence in the carotid arteries but also reported in the vertebrobasilar circulation (0.3-0.5%) [6]. Additionally, FFT can be found in the aortic arc, further emphasizing its diverse localization and potential clinical implications.

Regarding its natural history, FFT is associated with a high risk for stroke recurrence, and its potential outcomes include progression to occlusion, distal embolization, stabilization, or disappearance [5]. The optimal management is still unclear, with doubts concerning the duration and choice of antithrombotics, the benefits and risks of recurrence associated with intravenous thrombolysis, and the timing and efficacy of interventional treatments such as carotid artery stenting (CAS) or endarterectomy [4-5].

Here, we present a case series of eight patients with acute ischemic stroke in whom an FFT was identified and managed using medical or interventional approaches, leading to partial or complete thrombus resolution. The aim of this study is to describe the clinical, radiological, and etiological characteristics of FFT, report the therapeutic strategies applied and their outcomes, and review the literature to highlight the ongoing debate regarding optimal management strategies for symptomatic FFT.

## Materials and methods

Study population

We conducted a retrospective study over a 17-month period (2023-2024) at Ibn Rochd University Hospital. Patients admitted to the neurovascular unit during the acute phase of ischemic stroke were identified from the neurological emergency admission register. Inclusion criteria encompassed patients with ischemic stroke confirmed by MRI. Additionally, CTA and/or DUS of the supra-aortic trunks were performed to identify the presence of an FFT within the cervicocephalic arteries or the aortic arch. Patients diagnosed with lacunar strokes were excluded from the study.

Data collection

A dedicated database was created to record demographic and clinical data, including age, sex, and cardiovascular risk factors such as diabetes, hypertension, dyslipidemia, smoking, toxic consumption, and sleep apnea syndrome (SAOS). Additionally, past medical history and previous cardiovascular events (stroke, coronary artery disease, and peripheral vascular disease) were documented. The current treatment regimen, including the use of antiplatelet agents and anticoagulants, was also recorded.

**Figure 1:**
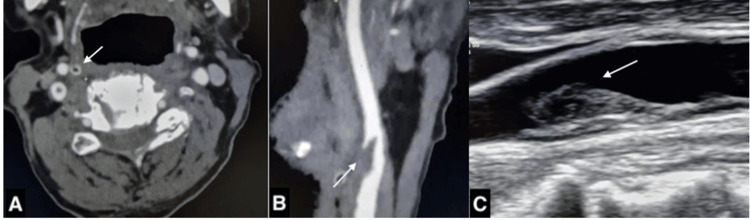
(A) The “donut sign” in a cross section (B) a free-floating thrombus attached to the carotid bulb and extending distally. (C) Carotid free-floating thrombus on a duplex ultrasound as a hyperechogenic formation arising proximally from the carotid wall and extending free to the arterial lumen distally

All patients underwent brain MRI, confirming ischemic stroke, with data collected on the affected vascular territories, radiological stroke patterns, and cervical vascular imaging findings. CTA and DUS were analyzed for the presence of the “donut sign”, defined as an intra-arterial filling defect completely surrounded by luminal contrast medium in at least two sequential axial slices, with proximal wall adherence (Figure 1A). The "finger sign", observed on sagittal views, describes an elongated, tapering thrombus projecting into the arterial lumen, with proximal wall adherence, resembling a digit, and is indicative of an FFT (Figure 1B).

Further investigations included cardiac assessment (electrocardiogram, transthoracic echocardiography) and comprehensive blood tests, including complete blood count (CBC), coagulation panel, electrolytes, and lipid panel. In cases where initial investigations were inconclusive, additional tests such as COVID-19 serology, autoantibody screening, thrombophilia panel, and thoraco-abdominopelvic CT scan were performed to rule out underlying malignancies.

Management strategies were classified into intravenous thrombolysis, antithrombotic therapy (antiplatelet or anticoagulant), and surgical intervention. Medical treatment was categorized into two groups: antiplatelet therapy, anticoagulant therapy, which included low-molecular-weight heparin (LMWH) or direct oral anticoagulants (DOACs).

Follow-up data included clinical evolution, recurrence of ischemic or transient ischemic strokes, and the modified Rankin Scale (mRS) at one and three months. Imaging follow-up was performed using CTA or DUS of the supra-aortic trunks at the same time points to assess thrombus resolution or persistence.

## Results

Among 940 recorded cases of ischemic stroke, eight patients (0.8%) were diagnosed with a free-floating thrombus (FFT). The mean age was 50.6 years (range: 31-70 years), with an equal sex ratio (four men, four women). Data on cardiovascular risk factors are detailed in Table 1. The mean NIHSS score at admission was 11.5 (range: 0-20). 

**Table 1:** Clinical Characteristics, Management and Outcomes of Included Patients (n = 8) NIHSS: National Institutes of Health Stroke Scale; MCA: middle cerebral artery; ACA: anterior cerebral artery; VB: vertebrobasilar territory. SAOS: sleep apnea obstructive syndrome; DOAC: direct oral anticoagulant; mRS: Modified Rankin Score.

Patient	1	2	3	4	5	6	7	8
Age	49	48	41	47	31	70	57	61
Sex	H	F	F	H	F	H	F	H
Stroke risk factors	Hypertension, smoking	Hypertension, dyslipidemia, smoking, SAOS, prior stroke	Smoking, SAOS	Hypertension, obesity, prior stroke	Cannabis use, smoking	Hypertension, diabetes, dyslipidemia, obesity, SAOS	Obesity, SAOS	Hypertension
Initial NIHSS	20	15	14	18	20	3	2	0
Vascular territory	MCA	MCA+VB	MCA	MCA	MCA	MCA+VB	VB	MCA
Etiologies	Carotid dissection	Atherosclerosis	Carotid web	Atherosclerosis	Carotid Web	Atherosclerosis	Atherosclerosis	Atherosclerosis
Management in the acute phase	Enoxaparin and switch to aspirin	Enoxaparin + aspirin + statins	Enoxaparin and switch to DOAC	Enoxaparin and endarterectomy	Aspirin (malignant infarct)	Enoxaparin + statins	Enoxaparin + aspirin + statins	Enoxaparin + aspirin
mRS 1 month post stroke	5	5	4	5	5	2	3	0
mRS 3 months post stroke	3	3	2	3	3	1	1	0
Thrombus evolution after 3 months	Incomplete thrombus regression	Incomplete thrombus regression	Complete thrombus regression	Complete thrombus regression	Complete thrombus regression	Incomplete thrombus regression	Incomplete thrombus regression	Complete thrombus regression

**Figure 2:**
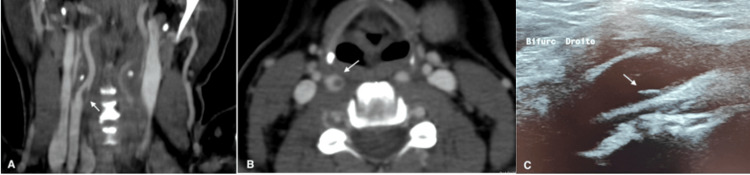
(A) Cross-sectional computed tomography angiography (CTA) image of Patient 3, revealing a carotid web accompanied by a free-floating thrombus within the internal carotid artery lumen. (B) Axial CTA view highlighting the characteristic “donut sign” . (C) Duplex ultrasound image obtained post-thrombus resolution, clearly delineating the prominent carotid web projecting into the arterial lumen.

Regarding stroke localization, seven patients presented with a middle cerebral artery (MCA) infarction, including two with additional vertebrobasilar involvement, while one patient had an isolated vertebrobasilar infarct. All patients underwent combined CTA and DUS, which confirmed the presence of an FFT. The thrombus was located in the cervical internal carotid artery (n=3), carotid bulb (n=3), brachiocephalic artery (n=1), and vertebral artery (V0 segment) (n=1). The identified underlying etiologies included atherosclerosis, carotid dissection, and carotid web (Figure 2). 

**Figure 3:**
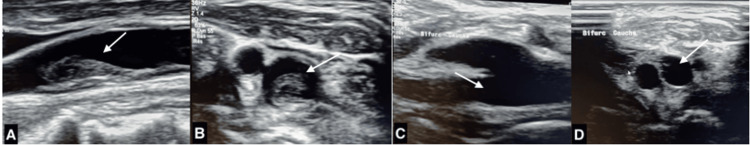
(A) Axial and (B) sagittal duplex ultrasound views illustrating a free-floating thrombus in the carotid artery of Patient 4 prior to endarterectomy, with the characteristic “donut sign” evident. Postoperative images (C) and (D) demonstrate complete resolution of the thrombus following surgical intervention.

Concerning management, all were treated medically, with three patients receiving enoxaparin and three others receiving a combination of anticoagulation and antiplatelet therapy, following confirmation of an atherosclerotic origin. One patient was placed on aspirin alone due to a malignant cerebral infarction requiring decompressive craniectomy. One patient underwent carotid endarterectomy 20 days post-stroke following a recurrent ischemic event (Figure 3).

During follow-up, one patient experienced a recurrent stroke, leading to surgical intervention. The modified Rankin Scale (mRS) at one and three months varied according to age and initial clinical severity. At three months, follow-up imaging showed complete thrombus resolution in four cases. 

Table 1 provides a comprehensive summary of the study population, including demographic characteristics, stroke risk factors, stroke severity, vascular territories, underlying etiologies, acute phase management, functional outcomes at one- and three-months post-stroke, and thrombus evolution over time.

## Discussion

Our study identified FFT in 0.8% of ischemic stroke cases, a rate consistent with previous reports. In contrast to the male predominance often described in the literature [2], we observed an equal sex distribution (four men and four women) and a slightly younger mean age (50.6 years vs. 57.6 years reported in previous studies). Atherosclerosis was the most common etiology [4], in line with existing data, followed by carotid web, a structural abnormality that promotes local blood stasis. Although traditionally considered rare, carotid web is increasingly recognized as an important cause of cryptogenic ischemic stroke, particularly among young African populations, where a higher prevalence has been reported [7]. Its mechanism involves artery-to-artery embolism due to localized blood stasis and thrombus formation [8]. Given its association with large vessel occlusions and severe stroke presentations, heightened diagnostic awareness is crucial, especially in our population. Other identified causes included hypercoagulable states (malignancy, thrombophilia, inflammatory conditions) and carotid dissection.

In our study, FFT was predominantly localized in the extracranial internal carotid artery, reinforcing its high frequency in this location, as previously described in the literature [9]. However, FFT in the intracranial internal carotid artery remains underdiagnosed, primarily due to the widespread reliance on cervical DUS, which does not assess intracranial vessels. This highlights the importance of complementary vascular imaging, particularly CTA, to improve detection rates [6]​​​​​​​. FFT in the posterior circulation remains poorly documented, with only isolated case reports and small case series available. The clinical and imaging characteristics of vertebrobasilar FFT are less well understood, yet one patient in our study presented with FFT in the vertebral artery (V0 segment). Given its association with higher morbidity and mortality, early detection of posterior circulation FFT is particularly crucial [10]​​​​​​​​​​​​​​.

The diagnostic approach to FFT has evolved significantly over time. Previously, digital subtraction angiography (DSA) was considered the gold standard, but its invasive nature and associated risks have led to its gradual replacement by non-invasive techniques such as CTA and DUS [5-11]​​​​​​​​​​​​​​. In our study, all patients underwent CTA and DUS, both for initial diagnosis and follow-up monitoring. Several imaging markers have been described to enhance FFT detection. The "donut sign", characterized by a hypoechoic ring on DUS or a contrast-surrounded thrombus on CTA, is one of the most reliable indicators of FFT [12]​​​​. Additionally, the "finger sign" on sagittal CTA, representing an elongated thrombus protruding into the arterial lumen, was identified in multiple cases in our study [5]​​​​​​​. These findings were systematically assessed to confirm FFT resolution during follow-up [13].​​​​​​​

The optimal treatment for FFT remains controversial, with no established consensus on management. The choice between antithrombotic therapy and surgical intervention depends on multiple factors, including thrombus size, mobility, and underlying etiology. Intravenous thrombolysis was not administered to any of our patients, reflecting the ongoing debate regarding its safety in FFT-associated ischemic stroke. In fact, its indication in this context remains highly controversial. A systematic review suggested that patients receiving IV thrombolysis had an increased risk of early stroke recurrence within the first 24 hours, compared to those who did not receive thrombolysis. Given the potential for thrombus fragmentation and embolization, the risks of intravenous thrombolysis in this setting may outweigh the benefits, particularly in large, mobile thrombi [5]​​​​​​​.

Several studies have suggested that antithrombotic therapy (anticoagulation and/or antiplatelet therapy) combined with statins enhances thrombus resolution [14]​​​​​​​. Our findings support this observation, as three patients received enoxaparin, three were treated with a combination of anticoagulation and antiplatelets, and one received aspirin alone due to malignant infarction requiring decompressive craniectomy. Notably, three patients showed complete thrombus resolution at three months, reinforcing the effectiveness of medical therapy in select cases. Recent literature suggests that no significant difference in clot resolution exists between anticoagulation and antiplatelets when used independently [14]​​​​​​​. However, combined therapy (anticoagulants, antiplatelets, and statins) appears to have a superior effect, particularly in atherosclerotic FFT, as supported by Ní Chróinín et al. [15] and also the meta-analysis published by Jayyusi et al. [16], who found that statin administration significantly improves stroke outcomes​​​​​​​. This is likely due to statins' pleiotropic effects on plaque stabilization and thrombus regression. In fact, according to the 2023 recommendations of the European Journal of Vascular and Endovascular Surgery (ESVS) concerning the management of FFT, anticoagulation should be used in patients with symptomatic FFT [17]​​​​​​​.

Only one patient in our cohort underwent carotid endarterectomy following recurrent ischemic stroke, reflecting the ongoing debate on the role of surgery in FFT management. Bhatti et al. found no clear superiority between medical and surgical approaches [2]​​​​​​​, while a study by Buchan et al. reported higher recurrence rates in surgically treated patients (37.5%) compared to medically managed patients (21.4%) [6]​​​​​​​. Similarly, Jayyusi et al. suggested that while procedural intervention in FFT management may achieve higher revascularization rates and potentially reduce stroke recurrence, it is associated with increased mortality and complication risks compared to medical therapy [16]​​​​​​​. In the Buchan cohort, 11 of 16 surgical patients underwent intervention within 27 hours of diagnosis, suggesting that early surgery may increase the risk of thrombus fragmentation and embolization [6]​​​​​​​. A French study further supported this theory, demonstrating that delaying surgery to 12 ± 5.5 days reduced recurrent events compared to early surgical intervention (3 ± 0.3 days in the Buchan series). Our findings align with these observations, as our surgically treated patient underwent delayed carotid endarterectomy, minimizing the risk of early embolic complications [9].​​​​​​​

Given these considerations, an initial anticoagulation-based approach, followed by delayed surgery if follow-up imaging shows persistent FFT or residual stenosis, appears to be a cautious and optimal strategy. The notion that immediate surgical intervention should be reconsidered is further reinforced by data showing that 86% of medically treated patients achieve complete FFT resolution without further neurological deterioration. Additionally, emerging evidence suggests that a combined approach-initial anticoagulation followed by delayed surgical intervention-could be beneficial, although randomized trials are still lacking to establish the optimal timing and risk-benefit balance [9].

This study has several limitations. First, the small sample size means that our findings are purely descriptive and cannot be generalized beyond the observed cases. Second, this study was monocentric, conducted in a single institution, which may introduce selection bias. Third, the retrospective design might have led to incomplete data collection or missing clinical and radiological information.

## Conclusions

In this case series, the management of FFT required an individualized approach. We observed that anticoagulation, with or without antiplatelet therapy, was effective in achieving thrombus resolution in most cases. Immediate endarterectomy was associated with a risk of embolization, which supports a stepwise strategy of initial anticoagulation, followed by surgery only in cases of persistent significant stenosis or recurrent events. These findings should be interpreted within the context of our small, single-center series. Further multicenter studies and long-term follow-up are needed to confirm these results and better define standardized management strategies.
